# Pulmonary function tests and rehabilitation in 2012: beyond sitting position

**DOI:** 10.1186/2049-6958-7-10

**Published:** 2012-06-20

**Authors:** Federica Gamna, Roberto Torchio

**Affiliations:** 1 Physical Medicine and Rehabilitation, AOU S. Luigi, Gonzole Region 10, Orbassano, Turin, Italy; 2 Cardiorespiratory Pathophysiology Department, Respiratory Function and Sleep Laboratory, AOU S. Luigi, Gonzole Region 10, 10043, Orbassano, Turin, Italy

## 

The benefits of pulmonary rehabilitation (PR) programs in patients with chronic obstructive pulmonary disease (COPD) are well established today [1]. PR programs improve exercise tolerance and health related quality of life; nevertheless, the role of inspiratory muscle training in rehabilitation programs remains controversial [2]. This is particularly due to the absence of clear data on the effects of inspiratory muscle training on outcomes in patients with COPD such as exercise capacity, functional exercise capacity, and dyspnea during activities of daily living.

In the present issue of M*ultidisciplinary Respiratory Medicine* Ki-song Kim & coll [3] present data about the effects of breathing maneuver and posture on muscle activity of inspiratory accessory muscles in COPD patients, concluding that the different involvement of accessory respiratory muscles can be easily studied in these patients, making it possible to monitor respiratory load during pulmonary rehabilitation. Though data on the correlation between dyspnea and breathing pattern were lacking, the study considered particular real life situations as pursed lip breathing and bracing arms position.

It is well known that posture during spontaneous quiet breathing influences not only thoraco-abdominal kinematics but also gas exchange and the cardiovascular system. Hence, in recent years functional studies and respiratory rehabilitation procedures have taken posture into account as a strategic way to understand and treat cardiorespiratory disability.

PR in the past was performed with good results for patients but often subjects were studied in seated position only, with spirometry measuring forced expiratory volume in 1 s. In this way the evidence of cardiorespiratory function improvements after treatment was lacking. Moreover PR was considered like a flying bumble-bee [4] (according to conventional aerodynamic principles, bumble bees cannot fly) and this paradox was resolved only when it became clear that the bumblebee’s flight is more like that of a helicopter than an aeroplane. For PR the apparent paradox was resolved when it became clear that patients with chronic respiratory disease experience substantial morbidity from secondary impairments, such as peripheral muscle, cardiac, nutritional and psychosocial dysfunction, which can be mitigated by PR. Only different approaches considering complex interaction between cardiovascular, respiratory and muscular systems in real life situations are able to clarify single patient disability and document physiological improvements.

As an example of real life, very recently [5] respiratory postural changes were investigated by means of new technologies, such as optoelectronic plethysmography, in normal humans and neuromuscular patients with regard to early impairment detection and in order to clarify breathing in particular conditions as supine position or wheel chair sitting. Real life data related to daily occupations can guide prescription and follow up in PR.

Another example commonly present in real life measurement can be considered. In normal elderly subjects the arterial oxygen pressure is lower in supine than in sitting position, because closing capacity exceeds functional residual capacity with increased inhomogeneity of ventilation-to-perfusion due to airway opening and closure during quiet breathing [6]. This simple fact has clinical implications since in bedridden patients the P_a_O_2_ is frequently measured in blood samples taken from supine subjects, and compared to normal values obtained in seated subjects. Such comparisons are not valid because in the supine position the values of P_a_O_2_ tend to be lower than those in seated position, particularly in elderly subjects. The same applies to estimates of arterial oxygen saturation.

So any attempt to carefully understand functional respiratory mechanisms and respiratory muscle involvement in the real life procedures during PR maneuvers is important, also for the purposes of clarifying if the addition of inspiratory muscle training to general exercise training results in greater benefits than exercise training alone in COPD patients.
